# Immunomodulatory Effects of Hemagglutinin- (HA-) Modified A20 B-Cell Lymphoma Expanded as a Brain Tumor on Adoptively Transferred HA-Specific CD4^**+**^ T Cells

**DOI:** 10.1155/2014/165265

**Published:** 2014-02-16

**Authors:** Valentin P. Shichkin, Roman M. Moriev

**Affiliations:** ^1^Department of Immunology, University “Ukraine”, 23 Lvivska Street, Building 2, Room 301, Kyiv 03115, Ukraine; ^2^Department of Chemical and Biomolecular Engineering, Whiting School of Engineering, Johns Hopkins University, Baltimore, MD 21218, USA

## Abstract

Previously, the mouse A20 B-cell lymphoma engineered to express hemagglutinin (HA) antigen (A20HA) was used as a systemic tumor model. In this work, we used the A20HA cells as a brain tumor. HA-specific CD4^+^ T cells were transferred intravenously in a tail vein 5 days after A20HA intracranial inoculation and analyzed on days 2, 9, and 16 after the adoptive transfer by different methods. The transferred cells demonstrated state of activation as early as day 2 after the adoptive transfer and most the of viable HA-specific cells became anergic on day 16. Additionally, symptoms of systemic immunosuppression were observed in mice with massive brain tumors at a late stage of the brain tumor progression (days 20–24 after the A20HA inoculation). Despite that, a deal of HA-specific CD4^+^ T cells kept the functional activity even at the late stage of A20HA tumor growth. The activated HA-specific CD4^+^ T cells were found also in the brain of brain-tumor-bearing mice. These cells were still responding to reactivation with HA-peptide *in vitro*. Our data support an idea about sufficient role of both the tumor-specific and -nonspecific mechanisms inducing immunosuppression in cancer patients.

## 1. Introduction

While gliomas are the most common primary malignant tumors of brain, lymphomas also contribute significantly in frequency of primary central nervous system (CNS) tumors, especially in patients receiving an immunosuppressive therapy.

Induction of immune response to tumors located in brain is limited by blood brain barrier (BBB) that bounds access of T cells to the CNS as well as failure of brain environment to activate infiltrating T cells fully [1, 2]. Although the BBB may pose an obstacle for migration of naive T cells into brain, there is considerable evidence that activated T cells are able to pass through the BBB and enter brain for antigen surveillance [3–5]. Particularly, circulating CD8^+^ T lymphocytes activated outside brain with tumor-specific antigens may enter into the brain and develop a local cytotoxic response against tumor [2, 6–11]. Certain evidences indicate that CD4^+^ T-helper (Th) cells can also enter into brain [11, 12], and they are an equally critical component of antitumor immune response [11, 13–17]. However, tumor-specific CD4^+^ T cells can be rendered tolerant (anergic) when they encounter antigen in absence of a costimulatory signal [18–20]. Anergic CD4^+^ T cells are neither deleted nor altered with regard to levels of T cell receptor for antigen (TCR) and coreceptor molecules, such as B7, but are refractory to an antigenic stimulus that would activate naive T cells [21, 22]. Though tumor-specific CD4^+^ Th cells are necessary for generation of potent antitumor immunity, there still are little known about fate of these Th cells during a lymphoma progression in brain.

Mouse A20 B-cell lymphoma modified with influenza hemagglutinin (HA) gene to express HA antigen (A20HA) was developed as an experimental system allowing the quantitative determination of systemic tumor progression effects on population of naive TCR clonotypic CD4^+^ Th cells that is specific for HA antigen in terms of their proliferation versus depletion and state of activation versus anergy [4, 20, 21, 23]. A20 cells express high levels of MHC class I and class II molecules as well as constitutively low levels of T cell costimulatory molecules CD80 (B7-1) and CD86 (B7-2), and they are able to present both exogenous and endogenous antigens [20]. This tumor behaves *in vivo *similar to many forms of human B-cell lymphoma. Systemic intravenous (i.v.) injection of A20 cells results in spread to spleen, mesenteric lymph nodes, and liver. At late stages, the cells can also be found in bone marrow and blood [16]. Thus, this approach allows for determining changes in phenotype and function of adoptively transferred HA-specific CD4^+^ T cells including their proliferation, depletion, state of activation, and anergy following exposure to HA antigen in tumor-bearing mice [16, 20, 21, 24].

In this study, we used A20HA cells as a brain tumor experimental model to evaluate immunomodulatory effects of a brain lymphoma on adoptively transferred HA-specific CD4^+^ T cells. We have established survival rate of A20HA brain-tumor-bearing mice and demonstrated possibility of adoptively transferred CD4^+^ T cells to the tumor-specific activation *in vivo* as well as development of the tumor-specific anergy in process of the brain tumor progression. We also demonstrated that although the tumor-specific anergy as well as symptoms of systemic immunosuppression is developed in A20HA brain tumor-bearing mice, there still exist CD4^+^ Th cells responding to HA-specific restimulation even at late stages of the brain tumor progression, and the activated HA-specific T cells could be found in the brain. These results provide important insight into continued efforts to develop combined chemoimmunotherapy modalities for patients with brain lymphomas, which could include systemic adoptive transfer of tumor-specific T cells and DNA vaccination as well as local cytokine and chemotherapy delivery [11, 17, 25–27].

## 2. Materials and Methods 

### 2.1. Mice

BALB/c female mice, 4- to 6-week old, were obtained from the National Institutes of Health (Frederick, MD). TCR transgenic mice expressing *αβ*TCR specific for influenza HA peptide (amino acids 110–120) were kindly provided by Prof. H. Levitsky. These mice were crossed to a BALB/c background for more than ten generations before using and were heterozygous for the transgene (HA MHC II^+/−^ Thy1.1^+/−^ 6.5^+/−^). All experiments involving the use of mice were performed in accordance with protocols approved by the Animal Care and Use Committee of the Johns Hopkins University School of Medicine.

### 2.2. Antibodies

Biotin-labeled rat anti-TCR antibodies 6.5, FITC-conjugated goat anti-mouse CD4, and FITC-conjugated anti-CD44 antibodies were purchased from Caltag (South San Francisco, CA). Cy-Chrome-conjugated rat anti-mouse CD4 (RM4-5), FITC-conjugated rat anti-mouse CD44 (IM7), and PE- or perCp-conjugated mouse anti-rat/mouse Thy1.1 (OX-7) antibodies were purchased from PharMingen (San Diego, CA).

### 2.3. A20HA Cell Line

A20HA cell line (BALB/c background) was created by electroporation and plasmid transfection of A20 cells with HA gene [16] and was kindly provided by Prof. H. Levitsky. The cells were cultured at 37°C in 5% CO_2_ atmosphere with neomycin analogue G418 (400 *μ*g/mL) in RPMI 1640 media supplemented with fetal calf serum (10%), penicillin/streptomycin (50 U/mL), L-glutamine (2 mM), and 2-mercaptoethanol (50 *μ*M) as described [20]. A stereotactic technique was used for intracranial (i.c.) injection of A20HA cells in the left parietal lobe of brain of BALB/c mice (1 × 10^4^ or 5 × 10^4^ cells) in a volume of 2 *μ*L Hanks Buffered Salt Solution (HBSS) as described [6]. For systemic i.v. inoculation, A20HA cells (1 × 10^6^ in 200 *μ*L HBSS) were injected into a tail vein as described [20].

### 2.4. Adoptive Transfer

Lymph nodes and spleens were collected from TCR transgenic BALB/c mice from estimate one donor to four recipients [21], homogenized in RPMI-1640 media, and passed over nylon mesh. After lysis of red cells, lymphocytes were washed in HBSS and percentage of HA-specific T cells positive for CD4 and clonotypic TCR (Thy1.1^+^ and/or 6.5^+^) was determined by FACS. The cells were injected i.v. into a tail vein of recipient BALB/c mice (three mice per group) such that a total of 2.5 × 10^6^ CD4^+^ TCR clonotypic T cells [20] were transferred to each recipient 5 days after the tumor challenge.

### 2.5. Mouse Survival Experiments and Histopathology

All mouse survival experiments following A20HA i.c. challenge included five mice per group. Each experiment was repeated at least twice. Animals were monitored for any signs of neurotoxicity and autopsied to confirm that death was due to a brain tumor. Brains, spleens, livers, thymuses, and lymph nodes were collected on days 7, 14, and 21 after the tumor inoculation, fixed in 10% formalin, blocked in paraffin, and sectioned in 10 *μ*m sections. Each section was then stained with eosin and hematoxylin and analyzed under a light microscope. Photomicrographs were taken with 10-, 20-, and 40-fold magnifications or direct scanning.

### 2.6. RT-PCR and *In Vitro* Cell Cultures for Metastases

Spleens, lymph nodes, and livers were collected from three mice per group on days 14 and 21 after i.c. (5 × 10^4^ cells) and i.v. (1 × 10^6^ cells) A20HA inoculation. After red cell lysis, cells were washed in HBSS and RNA was extracted from 2 × 10^6^ cells using a QIAGEN RNA extraction kit. Reverse transcription was performed with the SuperScript First-Strand Synthesis System (Invitrogen). cDNA amounts were analyzed by RT-PCR with Taqman System (Applied Biosystems). Each sample was assayed in triplicate for HA together with the internal reference, HPRT, using Taqman Universal PCR Master Mix and ABI Prism 7700 Sequence Detection System (Applied Biosystems). The relative HA mRNA frequencies were determined by normalization to HPRT. cDNA from BALB/c splenocytes was used as a negative control. The primer sequences for HA were 5′-CGCCGGATGGCTCTTG-3′ (forward) and 5′-ACAATGTAGGACCATGATCTCACTG-3′ (reverse). The HA-specific probe sequence was 5′-6FAMAAACCCAGAATGCGACCCACTGCTTTAMRA-3′. For *in vitro* cell culture assay, 2 × 10^6^ cells per sample were added to 6-well plates with 5 mL of the G418 selection media, and cell growth was monitored for 7 days.

### 2.7. Flow Cytometry

Lymphocyte suspensions were prepared as described above and washed with FACS buffer, and 1 × 10^6^ cells per samples were stained in 20 min with a standard procedure for three-color flow cytometry. Fifty thousand gated events were collected on a FACScan (Becton Dickinson, San Jose, CA) and CD4^+^CD44^+^Thy1.1^+^ T cells were analyzed with CellQuest software (Becton Dickinson). Background staining of a specified area from control BALB/c mice was usually lesser 0.01%.

### 2.8. DNA-HA Vaccination

A recombinant vaccinia virus encoding HA antigen from the 1934 PR8 strain of influenza virus was kindly provided by Prof. H. Levitsky. Virus HA-vaccine (HA-Vac) was expanded on HU-TK^−^ cells in presence of 5-bromo-2′-deoxyuridine (Sigma) at 25 *μ*g/mL. Virus was purified from the cellular lysate by sucrose banding and tittered by plaque assay on BSC-1 cells. HA-Vac (10^7^ PFU) was delivered intraperitoneally (i.p.) in 0.1 mL HBSS as described [20] on day 15 after the adoptive transfer.

### 2.9. *In Vitro* Cell Cultures for FACS, Proliferative, and ELISA Assays

A total of 1 × 10^6^ spleen cells or cervical lymphocytes extracted from recipient mice were incubated in round-bottom 96-well plates with 10 *μ*g/mL of MHC class II synthetic HA peptide. Cell cultures were harvested 72 h later and analyzed by FACS for CD4^+^CD44^+^Thy1.1^+^ T cells. For proliferative assay, the HA-stimulated cell cultures were pulsed finally 18 h with 1 *μ*Ci [^3^H]TdR, and radioactivity was measured with a liquid scintillation counter as described [20]. For ELISA (IFN-*γ* assay), the supernatants from HA-stimulated cell cultures were harvested after 72 h incubation, and IFN-*γ* concentrations were measured using the Quantikine M ELISA kit for murine IFN-*γ* according to the manufacturer's instruction (R&D Systems, Minneapolis, MN). Individual data points of all three assays represent the mean of triplicate wells from three mice per group.

### 2.10. Statistical Analysis

A paired *t*-test was used to compare values where appropriate. The values of *P* < 0.05 were considered statistically significant. Statistical analysis for mouse survival was performed using Kaplan-Meier survival and log-rank (Mantel-Cox) test. Statview 4.5 software (San Francisco, CA) was used for analysis.

## 3. Results and Discussion

### 3.1. A20HA Intracranial Growth and Survival Rate of A20HA  Brain-Tumor-Bearing Mice

**Figure 1:**
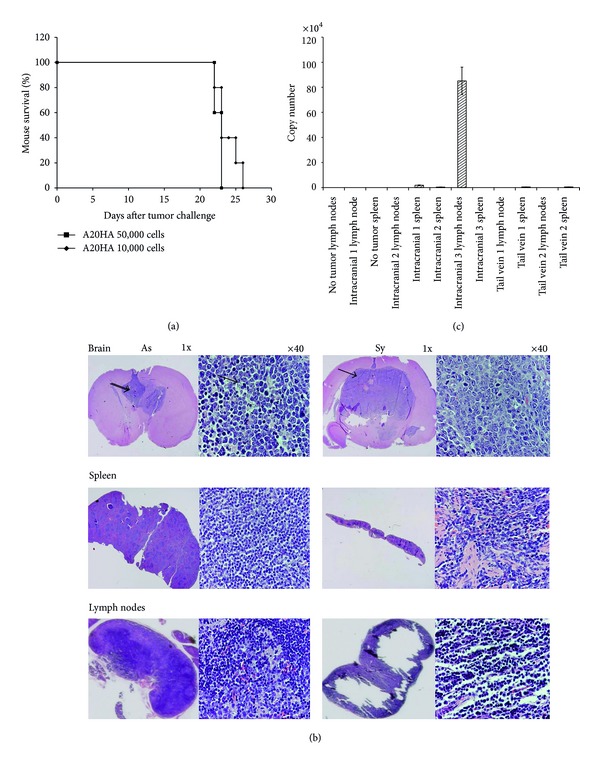
A20HA brain tumor growth in syngeneic BALB/c mice leads to fast death of mice and fatal depletion of spleens and lymph nodes in symptomatic mice. (a) Kaplan-Meier survival curve showing growth rates of A20HA B-cell lymphoma in brain after i.c. injection of 10,000 and 50,000 tumor cells (*n* = 5 in each group). A representative experiment among the three is shown. (b) Photomicrographs of eosin and hematoxylin stained coronal sections of the brains, spleens, and lymph nodes from (a) on day 21 after A20HA i.c. inoculation showing the forming of brain tumors (showed by arrows) and depletion of spleens and lymph nodes in asymptomatic (As) and symptomatic (Sy) mice that received 10,000 and 50,000 tumor cells, correspondingly. Magnifications ×1 and ×40. (c) RT-PCR of spleens and lymph nodes showing minor metastases of A20HA cells in lymph nodes on day 14 after i.c. A20HA injection (50,000 cells). Three individual mice in each group were analyzed. *P* < 0.05.

To assess the survival rate of A20HA brain-tumor-bearing mice, syngeneic BALB/c mice received i.c. injections of either 1 × 10^4^ or 5 × 10^4^ of A20HA cells. All mice that received 5 × 10^4^ cells died within 23 days of the treatment with a median survival of 22.5 days (Figure 1(a); *P* < 0.05). Mice that received 1 × 10^4^ cells died within 26 days with a median survival of 24 days (*P* < 0.05). Histological analysis of brains revealed that metastases were occasionally seen in the brain parenchyma distant from the injection site, and the tumor cells readily spread throughout the ventricles in the majority of animals (Figure 1(b)). The mice that showed such symptoms as untidiness, behavioral disorder, and weight loss (symptomatic mice) at the late stage of the tumor growth died in 1-2 days following these symptoms. Symptomatic mice had massive tumors at the injection site in contrast to mice which still had non of the above mentioned symptoms (asymptomatic mice). Rare infiltrates of lymphoid cells and massive necrotic areas, especially in symptomatic mice were also found (Figure 1(b)).

Thus, A20HA cells formed massive brain tumors in mice with 100% lethality within 23–26 days, and apparent metastasis outside the brain was not found by visual and microscopy study of all major organs and lymph nodes. This relates to other experimental and clinical observations concerning the preferentially localized growth of primary brain lymphomas [28–30]. However, A20HA cells were found in lymph nodes of the brain-tumor-bearing mice at the late stage of the tumor progression by means of RT-PCR (Figure 1(c); *P* < 0.05) as well as by cell culture analysis (data not shown).

Next, we found the fatal depletion of lymphoid organs in symptomatic mice versus asymptomatic ones. In particular, spleens were reduced in size and cell numbers at least by 5- to 10-fold in symptomatic mice versus asymptomatic ones, and massive areas of lymphocyte depletion in spleen and lymph nodes were found (Figure 1(b)).

### 3.2. HA-Specific Activation and Depletion of CD4^+^ T Cells

In tumor-bearing mice depletion of activated antigen-specific T cells and development of anergy (diminution or lack of immunity to the antigen) are two general mechanisms of T cell tolerance to tumors [31–34]. The state of activation or anergy in Th cells can be monitored by quantitative dynamics of CD4^+^ T cells and by level of CD44 expression that is increased under the activation [15, 16, 20, 21].

**Figure 2:**
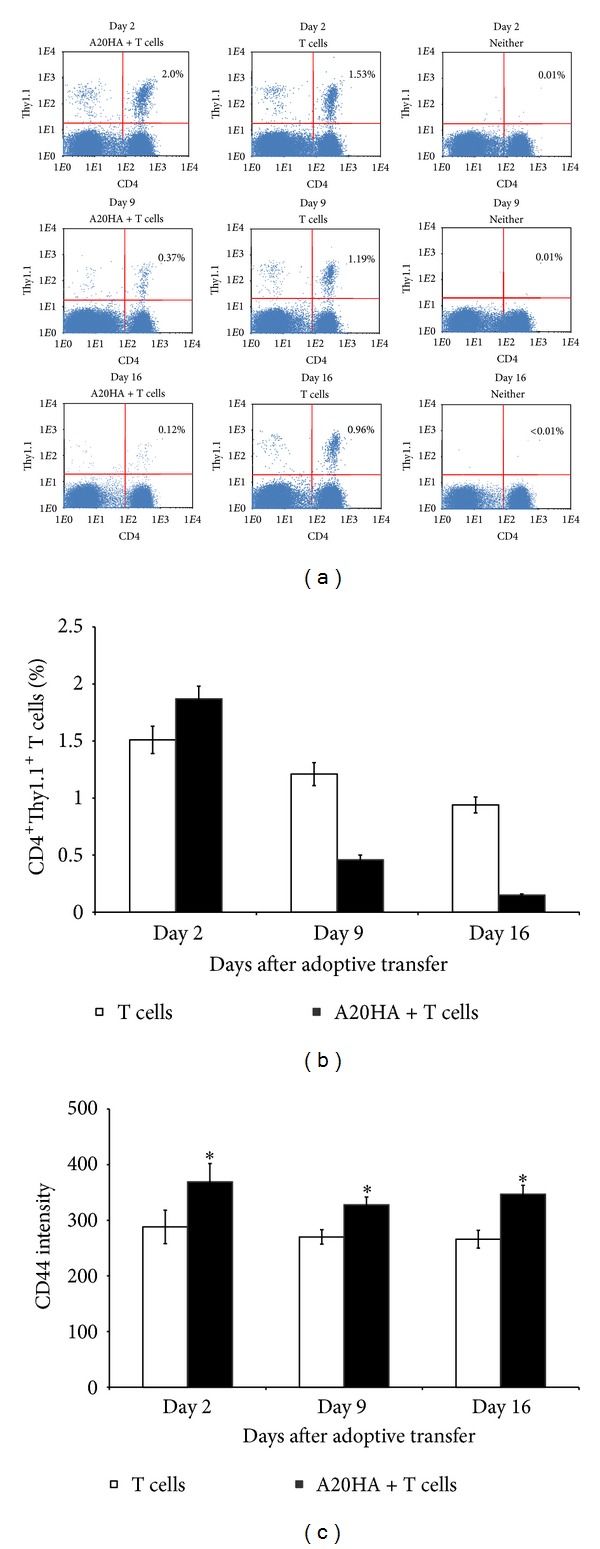
Adoptively transferred HA-specific CD4^+^Thy1.1^+^ T cells demonstrate early activation and following depletion in cervical lymph nodes of A20HA brain tumor-bearing mice. ((a), (b), (c)) Flow cytometry of CD4^+^Thy1.1^+^CD44^+^ T cells from cervical lymph nodes on days 2, 9, and 16 after adoptive transfer. HA-specific CD4^+^Thy1.1^+^ T cells were injected i.v. into A20HA brain tumor-bearing mice 5 days after i.c. tumor inoculation (A20HA + T cells; left column) and tumor-free control mice (T cells; middle column). Naive mice represented a negative control (Neither; right column). Each group included three mice. Cervical lymph nodes were analyzed individually from each mouse by three-color FACS assay. A representative experiment among three equivalents is shown. (a) Dot plots show gated HA-specific CD4^+^Thy1.1^+^ T cells (upper right quadrants; numbers in the quadrants indicate percent cells in each). Bars show (b) percent of HA-specific CD4^+^Thy-1.1^+^ T cells and (c) intensity of CD44 expression in the gated population of CD4^+^Thy-1.1^+^ T cells. Data are represented as the mean ± SD, *n* = 3. *P < 0.05.

In our experiments, cervical and inguinal lymph nodes as well as spleens from each mouse were analyzed by FACS with three-color staining for CD4^+^CD44^+^Thy1.1^+^ HA-specific T cells on days 2, 9, and 16 after the adoptive transfer (days 7, 14, and 21 after A20HA i.c. inoculation, resp.) (Figure 2). We observed that in contrast to tumor-free mice in the tumor-bearing mice both the percent of CD4^+^Thy1.1^+^ T cells (Figures 2(a) and 2(b); *P* < 0.05) and the level of CD44 expression by these cells (Figure 2(c); *P* < 0.05) were increased in cervical lymph nodes as early as day 2 after the adoptive transfer, and the increasing of CD44 expression was kept until day 16. At the same time, significant depletion of the transferred CD4^+^ T cells in cervical lymph nodes was found on day 9 and especially on day 16 (*P* < 0.05). The same quantitative dynamics of CD4^+^ T cells was observed also in inguinal lymph nodes and spleens (data not shown).

### 3.3. Response of HA-Specific CD4^+^ T Cells to Restimulation with HA Peptide *In Vitro*

The response of adoptively transferred HA-specific CD4^+^ T cells to restimulation *in vitro* with MHC class II synthetic HA peptide that is valuable for analysis the activation or anergic state in adoptively transferred T cells [20, 21] was evaluated. Mice were sacrificed on days 2, 9, and 16 following the adoptive transfer, and pools of lymphocytes from cervical and inguinal lymph nodes and spleens were analyzed by FACS before and after 3-day incubation with or without HA peptide. Proliferative response to HA peptide and levels of IFN-*γ* production *in vitro* were also measured in the samples as indexes of activation versus anergy [20, 21].

**Figure 3:**
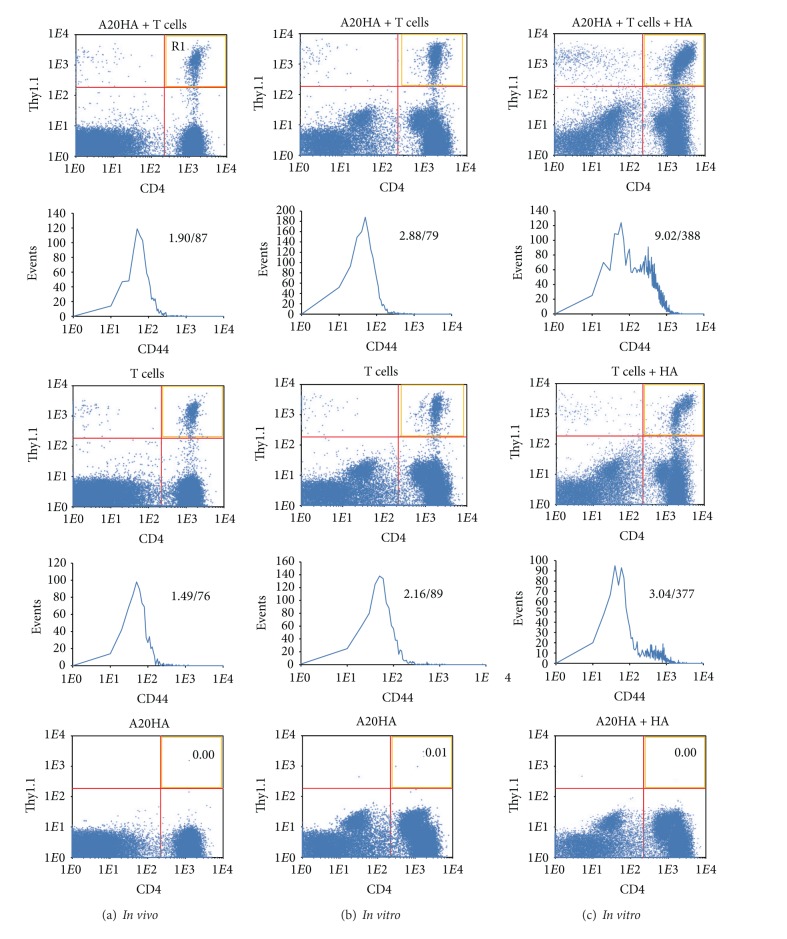
HA-specific CD4^+^Thy1.1^+^ T cells from cervical lymph nodes of A20HA brain tumor-bearing mice show increased response to restimulation *in vitro* with MHC class II synthetic HA peptide. ((a), (b), (c)) Flow cytometry of CD4^+^Thy1.1^+^CD44^+^ T cells from cervical lymph nodes on day 2 after adoptive transfer. HA-specific CD4^+^Thy1.1^+^ T cells were injected i.v. into A20HA brain tumor-bearing mice 5 days after i.c. tumor inoculation (A20HA + T cells; dot plots in the upper row) and a tumor-free control group (T cells; dot plots in the middle row). A20HA brain tumor-bearing mice in the absence of HA-specific CD4^+^Thy1.1^+^ T cells represented a negative control (A20HA; dot plots in the lower row). Cervical lymph nodes were pooled and analyzed for CD4^+^Thy1.1^+^CD44^+^ T cells (a) before incubation (*in vivo* column; right upper quadrants) and ((b), (c)) after 72 h incubation *in vitro* in triplicates (b) without (*in vitro* column; right upper quadrants) and (c) with (*in vitro* column; right upper quadrants) HA peptide. Histograms under the dot plots show the level of CD44 expression in the gated populations of CD4^+^Thy-1.1^+^ T cells (shown in the right upper quadrants). Numbers above histograms indicate percent of gated CD4^+^Thy-1.1^+^ T cells in the designed dot plots (left numbers) and intensity of CD44 expression by these cells (right numbers). One representative experiment of equivalent two is shown.

**Figure 4:**
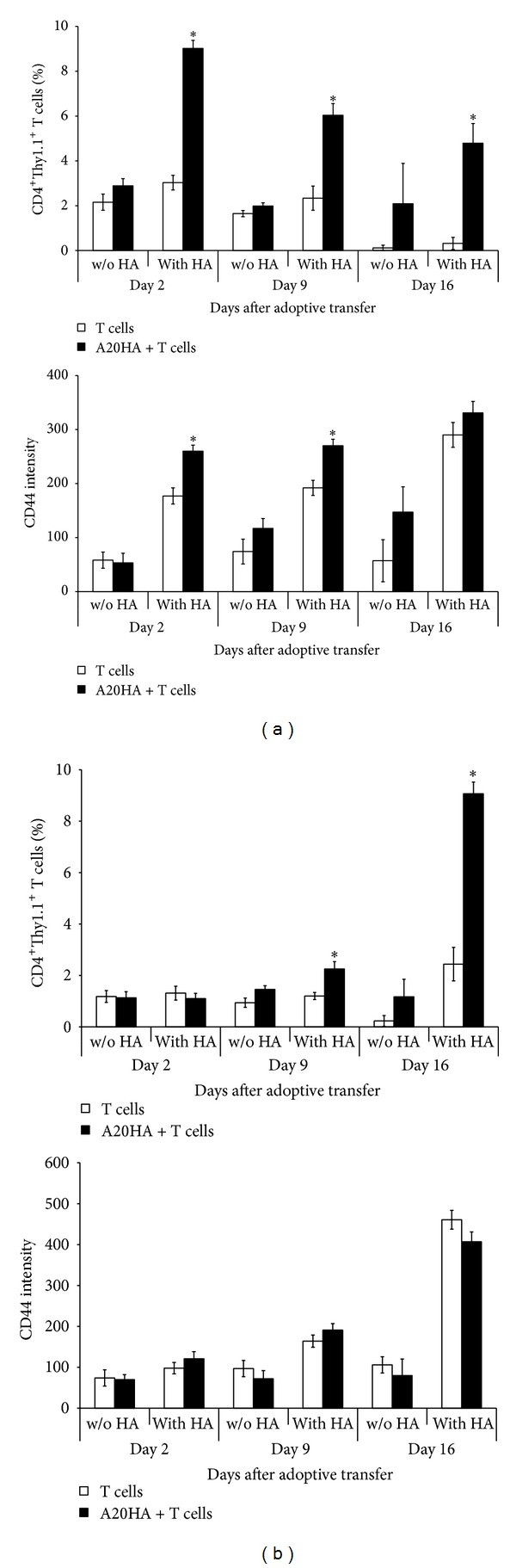
Response of HA-specific CD4^+^Thy-1.1^+^ T cells from cervical lymph nodes of A20HA brain tumor-bearing mice to HA peptide *in vitro* is reduced in process of the tumor progression and controversial to response of CD4^+^ T cells from spleen. ((a), (b)) Percent of CD4^+^Thy-1.1^+^ T cells (upper) and intensity of CD44 expression (lower) by cells isolated from (a) cervical lymph nodes and (b) spleens on days 2, 9, and 16 after the adoptive transfer. HA-specific CD4^+^Thy1.1^+^ T cells were injected i.v. into A20HA brain tumor-bearing mice 5 days after i.c. tumor inoculation (A20HA + T cells) and tumor-free control mice (T cells). A20HA brain tumor-bearing mice in the absence of HA-specific CD4^+^Thy1.1^+^ T cells represented a negative control (background staining of a specified area was lesser than 0.01%; data not shown). Cervical lymph nodes and spleens were pooled and analyzed by FACS for CD4^+^Thy1.1^+^CD44^+^ T cells before incubation and after 72 h incubation without (w/o) and with HA peptide. FACS data are presented as a percent of double positive cells, CD4 versus Thy1.1, and the level of CD44 expression in the CD4^+^Thy1.1^+^ T-cell population. One representative experiment of equivalent two is shown. *P < 0.05.

In strong correlation with experiments above, the cervical HA-specific CD4^+^ T cells were already specifically activated *in vivo* on day 2 after the adoptive transfer to A20HA brain tumor-bearing mice (Figure 3(a); *P* < 0.05). The cell incubation with HA peptide *in vitro* significantly increased both the expression of CD44 and number of HA-specific CD4^+^ T cells (Figure 3(c); *P* < 0.05). The declined response to the HA peptide was observed on days 9 and 16 after the adoptive transfer (Figure 4(a), upper; *P* < 0.05), though this response was still very strong until day 16 (*P* < 0.05). In contrast, increasing of CD44 expression in response to *in vitro* HA stimulation was even higher on day 16 than on days 2 and 9 (Figure 4(a), lower; *P* < 0.05). In control group of mice which were not injected with the tumor cells, response to HA peptide evaluated by changing of HA-specific CD4^+^ T-cell number was comparatively low, and increasing of CD44 expression was lesser than in the tumor-bearing mice (Figure 3, middle row; *P* > 0.05 and Figure 4(a); *P* < 0.05). In contrast to cervical lymph nodes, significant increase in both the percent of CD4^+^Thy-1.1^+^ T cells and the expression of CD44 in spleen was observed only on day 16 after the adoptive transfer (*P* < 0.05), and the number of CD4^+^Thy-1.1^+^ T cells was equivalent to that observed in cervical lymph nodes on day 2 (Figure 4(b); *P* > 0.05).

**Figure 5:**
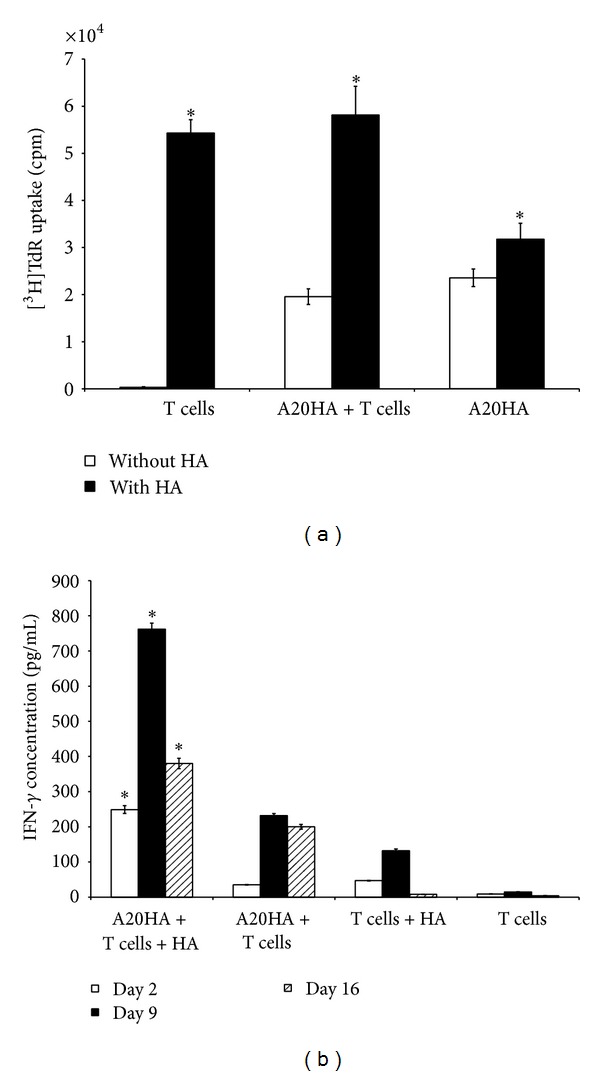
Proliferative response and production of IFN-*γ* by HA-specific CD4^+^Thy1.1^+^ T cells from A20HA brain tumor-bearing mice demonstrate nonanergic status of a part the adoptively transferred Th cells. HA-specific CD4^+^Thy1.1^+^ T cells were injected i.v. on day 5 after i.c. tumor inoculation into A20HA brain tumor-bearing mice (A20HA + T cells) and tumor-free control mice (T cells). A20HA brain tumor-bearing mice in the absence of HA-specific CD4^+^Thy1.1^+^ T cells represented a negative control (A20HA). Spleens and cervical lymph nodes were pooled and analyzed after 72 h incubation with or without HA peptide in triplicates. (a) Proliferative response of HA-specific CD4^+^ T cells from spleens of A20HA brain tumor-bearing mice to restimulation *in vitro* with HA peptide is not reduced. Cell proliferation was measured by [^3^H]TdR incorporation on day 16 after the adoptive transfer. (b) Production of IFN-*γ* by HA-specific CD4^+^ T cells from cervical lymph nodes of A20HA brain tumor-bearing mice induced by restimulation *in vitro* with HA peptide is maximal on day 9 and reduced on day 16 after the adoptive transfer. IFN-*γ* concentration was measured by ELISA. Data are represented as the mean ± SD, *n* = 3. *P < 0.05.

Proliferative response of HA-specific CD4^+^ T cells isolated on day 16 after the adoptive transfer from spleens of A20HA brain tumor-bearing mice to HA peptide *in vitro* was comparable with proliferative response of spleen cells from tumor-free mice that were injected with TCR transgenic T cells (Figure 5(a); *P* > 0.05) suggesting their nonanergic status. In contrast, proliferative response of spleen cells from A20HA brain tumor-bearing mice in absence of the TCR transgenic T cells was significantly lower (*P* < 0.05) which shows the specificity of A20HA stimulated activation of HA-specific CD4^+^ T cells *in vivo* and their specific restimulation with HA peptide *in vitro*.

The maximal level of IFN-*γ* production by HA-specific CD4^+^ T cells isolated from cervical lymph nodes the brain tumor-bearing mice was found on day 9 after the adoptive transfer both in presence of and without HA peptide (Figure 5(b)). However, the cells isolated from A20HA brain tumor-bearing mice on day 2 were already able to produce IFN-*γ* at the additional stimulation with HA peptide *in vitro*. IFN-*γ* production was impaired but still significant on day 16 (Figure 5(b); *P* < 0.05).

These data collectively show that the adoptively transferred HA-specific CD4^+^ T cells become activated already on day 2 after the adoptive transfer to A20HA brain tumor-bearing mice and reach the maximal activation on day 9 which correlates with increased IFN-*γ* production. At the late stage of the tumor progression significant depletion of the transferred CD4^+^ T cells (day 16) correlates with impaired level of IFN-*γ* production demonstrating the development of tumor-specific anergy. However, a sufficient part of functionally active tumor-specific CD4^+^ T cells is still kept (especially in spleen) even at late stages of A20HA brain tumor progression demonstrating their nonanergic status.

### 3.4. Response of HA-Specific CD4^+^ T Cells to HA Vaccination *In Vivo* and Restimulation with HA Peptide *In Vitro* in Symptomatic and Asymptomatic Mice

**Figure 6:**
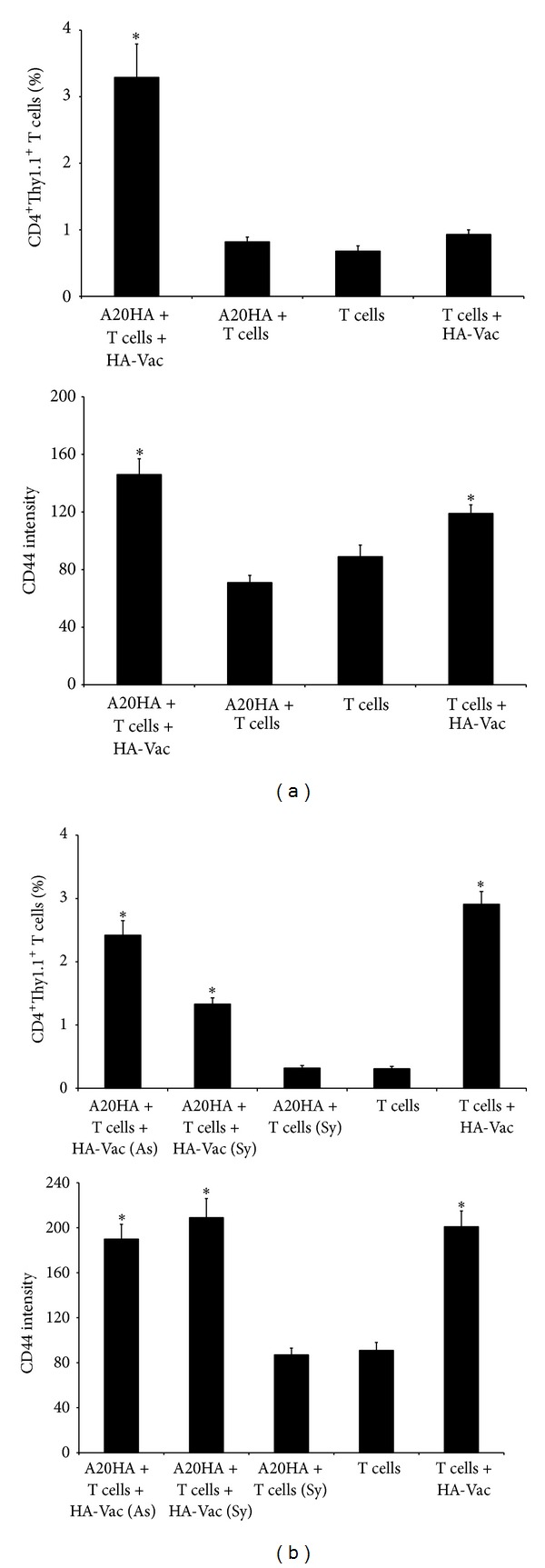
Virus HA vaccination at late stages of A20HA brain tumor progression stimulates activation of residual HA-specific CD4^+^ T cells *in vivo*. ((a), (b)) Percent of CD4^+^Thy-1.1^+^ T cells (upper) and intensity of CD44 expression (lower) by cells isolated from (a) cervical lymph nodes and (b) spleens from separated asymptomatic (As) and symptomatic (Sy) mice on day 18 after the adoptive transfer. HA-specific CD4^+^Thy1.1^+^ T cells were injected i.v. to A20HA brain tumor-bearing mice on day 5 after i.c. tumor inoculation. Virus HA-Vac was delivered i.p. on day 15 after the adoptive transfer. Individual cervical lymph nodes and spleens of asymptomatic and symptomatic mice (three mice per group) were analyzed by FACS for CD4^+^CD44^+^Thy1.1^+^ T cells. A20HA + T cells + HA-Vac, mice that received A20HA tumor cells, HA-specific CD4^+^Thy1.1^+^ T cells and virus HA-Vac; A20HA + T cells, mice that received A20HA tumor cells and HA-specific CD4^+^Thy1.1^+^ T cells; T cells + HA-Vac, mice that received HA-specific CD4^+^Thy1.1^+^ T cells and virus HA-Vac; T cells, mice that received only HA-specific CD4^+^Thy1.1^+^ T cells. Data are represented as the mean ± SD, *n* = 3. *P < 0.05.

Additionally, the state of anergy or activation in the adoptively transferred HA-specific CD4^+^ T cells was analyzed by their challenging with recombinant HA vaccinia virus (HA-Vac) *in vivo*. HA-Vac was injected on day 15 after the adoptive transfer and 3 days later (day 23 after A20HA inoculation) lymphocytes from cervical lymph nodes and spleens were analyzed by FACS for CD4^+^CD44^+^Thy-1.1^+^ HA-specific T cells separately in symptomatic and asymptomatic mice where it was possible. Though, the development of strong anergy in adoptively transferred T cells to this time of the tumor progression could be expected, the significant amount of CD4^+^ T cells responding to HA-Vac in cervical lymph nodes and spleens of asymptomatic mice was found (Figures 6(a) and 6(b), upper; *P* < 0.05), and the level of T-cell activation (CD44 expression) was comparable to mice without A20HA brain tumor (Figures 6(a) and 6(b), lower; *P* > 0.05). However, the symptomatic mice showed reduced response to *in vivo* HA-Vac measured by percentage change of CD4^+^Thy-1.1^+^ T cells in spleen compared to asymptomatic mice (Figure 6(b), upper; *P* < 0.05). Oppositely, a level of CD44 expression in CD4^+^Thy-1.1^+^ T-cell population was even higher in symptomatic mice versus asymptomatic ones (Figure 6(b), lower; *P* < 0.05). Though the late tumor-specific vaccination stimulated activation of remaining HA-specific CD4^+^ T cell, any prolongation of survival in this group of mice versus control groups bearing A20HA brain tumor only or A20HA brain tumor together with transferred CD4^+^ T cells was not observed (data not shown).

**Figure 7:**
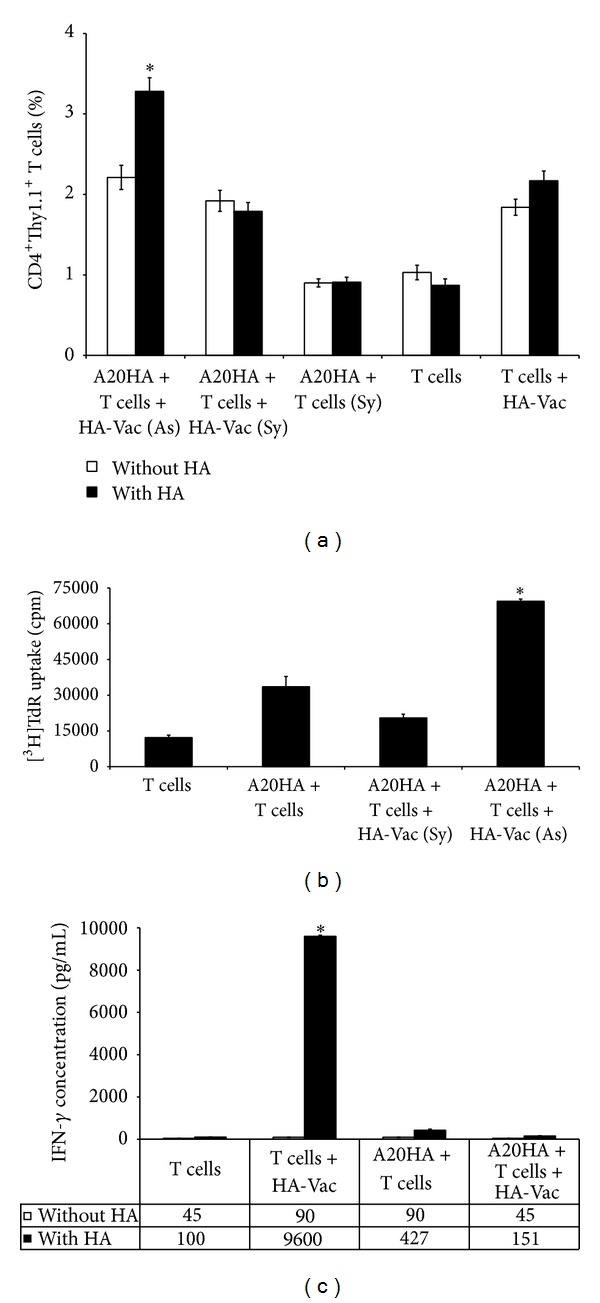
HA-specific CD4^+^ T cells from spleen of symptomatic mice versus asymptomatic are refractory to restimulation with HA peptide *in vitro* after HA vaccination *in vivo*. (a) Percent of HA-specific CD4^+^ Thy-1.1^+^ T cells measured by FASC, (b) proliferative response measured by [^3^H]TdR incorporation, and (c) concentration of IFN-*γ* in the supernatants measured by ELISA on day 18 after the adoptive transfer. HA-specific CD4^+^Thy1.1^+^ T cells were injected i.v. into A20HA brain tumor-bearing mice on day 5 after i.c. tumor inoculation. Virus HA-Vac was delivered i.p. on day 15 after the adoptive transfer. Individual spleens from asymptomatic (As) and symptomatic (Sy) mice were isolated 3 days later, and cell cultures were analyzed after 72 h incubation with or without HA peptide in triplicate wells. A20HA + T cells + HA-Vac, mice that received A20HA tumor cells, HA-specific CD4^+^Thy1.1^+^ T cells, and virus HA-Vac; A20HA + T cells, mice that received A20HA tumor cells and HA-specific CD4^+^Thy1.1^+^ T cells; T cells + HA-Vac, mice that received HA-specific CD4^+^Thy1.1^+^ T cells and virus HA-Vac; T cells, mice that received only HA-specific CD4^+^Thy1.1^+^ T cells. Numbers under axis in (c) show IFN-*γ* concentrations. Data are represented as the mean ± SD, *n* = 3. *P < 0.05.

Finally, the response of HA-specific CD4^+^ T cells from mice that received HA-Vac to restimulation with HA peptide *in vitro *was tested. Lymphocytes isolated from spleens of symptomatic mice on day 3 after HA vaccination were not able to respond to HA peptide in culture *in vitro* with increase of the percent of CD4^+^Thy-1.1^+^ T cells (Figure 7(a), A20HA + T cells + HA-Vac, Sy; *P* > 0.05). Proliferative response (Figure 7(b)) and production of IFN-*γ* (Figure 7(c)) in this group of mice were also significantly reduced compared to asymptomatic mice (*P* < 0.05). In contrast, though CD4^+^ T-cell response to HA peptide *in vitro* in absence of A20HA brain tumor was also insufficient (Figure 7(a), T cells + HA-Vac; *P* > 0.05), these cells were able to produce the significant quantity of IFN-*γ* compared to all other groups of mice (Figure 7(c); *P* < 0.05). Thereby, these data show the state of strong anergy in tumor-specific CD4^+^ T cells in symptomatic mice versus asymptomatic mice.

### 3.5. Migration of Activated HA-Specific CD4^+^ T Cells into Brain

**Figure 8:**
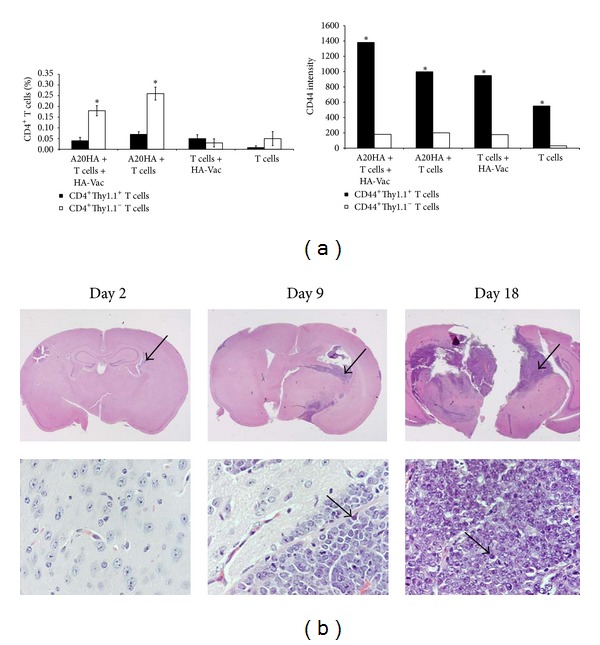
HA-specific activation stimulates migration of CD4^+^ T cells into the brain of A20HA brain tumor-bearing mice. (a) Percent (left bars) and CD44 intensity (right bars) of transgenic CD4^+^Thy1.1^+^ T cells and nontransgenic CD4^+^Thy1.1^−^ T cells from brains of A20HA brain tumor-bearing mice and A20HA tumor-free mice measured by FACS on day 18 after the adoptive transfer and HA vaccination. HA-specific CD4^+^Thy1.1^+^ T cells were injected i.v. on day 5 after A20HA i.c. inoculation, and virus HA-Vac was delivered i.p. at the same day. Brain pools of three mice in each group were analyzed by FACS 18 days later. A20HA + T cells + HA-Vac, mice that received A20HA tumor cells, HA-specific CD4^+^Thy1.1^+^ T cells, and HA-Vac; A20HA + T cells, mice that received A20HA tumor cells and HA-specific CD4^+^Thy1.1^+^ T cells; T cells + HA-Vac, mice that received HA-specific CD4^+^Thy1.1^+^ T cells and HA-Vac; T cells, mice that received only HA-specific CD4^+^Thy1.1^+^ T cells. (b) Photomicrographs of eosin and hematoxylin stained coronal sections of the brains on days 2, 9, and 18 after the adoptive transfer showing growth dynamics of A20HA B-cell lymphoma in BALB/c mice that received HA-specific CD4^+^ T cells and HA-Vac on day 5 after A20HA i.c. inoculation. Magnifications ×1 (upper row; arrows show the tumor areas) and ×40 (lower row, arrows show single lymphocytes in the tumor areas). *P < 0.05.

As known, activated T cells are capable of passing through the BBB and entering the CNS for antigen surveillance [3, 4]. The ability of adoptively transferred CD4^+^Thy1.1^+^CD44^+^ HA-specific T cells to penetrate into the brain and interact with the established A20HA brain tumor was evaluated by FACS on day 18 after the adoptive transfer and HA vaccination (Figure 8). The presence of a few number of highly activated (especially in group of HA-Vac mice) HA-specific CD4^+^CD44^+^Thy-1.1^+^ T cells into the brain of A20HA tumor-bearing mice as compared to non-HA-specific CD4^+^CD44^+^Thy-1.1^−^ T cells was found (Figure 8(a); *P* < 0.05). Single lymphoid cells were found also on histological slides of the brains with A20HA tumors in the tumor growth area at the all investigated days after the adoptive transfer along with macrophage and glial cells that are normal components of brain (Figure 8(b), lower). In spite of presence of highly activated HA-specific transgenic CD4^+^ T cells into the brain, HA vaccination did not show any apparent influence on the tumor growth in this experimental model (Figure 8(b), upper).

Taken together, data presented here demonstrate that though BBB limits access of peripheral lymphocytes to the brain as well as tumor cells to periphery, A20HA brain tumor can induce both the state of activation and anergy in adoptively transferred HA-specific CD4^+^ T cells. These data suggest that the activation of HA-specific T cells may be a result of challenging these cells with tumor HA antigen as outside the brain and within the brain. Although we did not observe significant infiltration of the brain tumors with foreign cells some numbers of the activated tumor-specific T cells could reach the brain. This possibility has been reported also with several experimental brain tumor models using local cytokine delivery that could support the growth of lymphocytes infiltrating a brain tumor and rescuing them from apoptosis [14, 35–37].

Other possibility for activation is capturing the soluble HA-antigen by dendritic cells outside the brain and presenting it to CD4^+^ T cells [17, 38]. Though, it was reported, the serum of systemic A20HA tumor-bearing mice does not contain tumor-associated HA antigens even at a late stage of tumor progression [20], some observations demonstrated migration of tumor antigens from brain to periphery [39]. In contrast, the interaction with minor metastases that were found at least in cervical lymph nodes at late stages of A20HA growth in brain by means of RT-PCR and cell culturing for mostly of HA-specific CD4^+^ T cells could lead to anergy because rejection of MHC class II tumor cells requires induction of tumor-encoded B7-1 and/or B7-2 costimulator molecules [40] and expression of these molecules by A20HA cells is low [20, 21]. This point is supported also by other experiments with increase of immune response against A20 B-cell lymphoma transfected with a gene of B7 costimulator molecule [41, 42] and development of tumor-specific tolerance in systemic A20HA tumor models [20, 21, 24, 43].

Moreover, it was demonstrated that cross-presentation of HA-antigen by bone marrow dendritic cells is dominant mechanism of the development of tumor-specific tolerance in systemic A20HA tumor models and direct contact of CD4^+^ T cells with tumor cells does not contribute significantly to that [20, 21, 24]. This mechanism of peripheral tolerization is not unique for lymphomas that express both MHC class I and MHC class II as well as costimulator B7-1 and B7-2 molecules and effective also for parenchyma self-antigens [31] and systemic tumors that express only MHC class I [17, 21, 44]. Moreover, regulatory CD4^+^CD25^+^ T cells that appear in periphery and can reach a malignant brain also may contribute to the immunosuppression [45]. Thus, these mechanisms together may contribute to the development of HA-specific anergy in the used experimental model.

A number of studies also support a hypothesis that tumors evade immunological rejection by inducing the state of global immunosuppression at a late stage of a tumor progression [20, 21]. This state has been clearly demonstrated in animals and patients with advanced tumors and is characterized by hyporesponsiveness to challenge with common recall antigen *in vivo* and diminished T cell function *in vitro* that correlates with specific alteration in T-cell signal transduction pathways [44]. Tumor cells can produce several factors such as TGF-*β*, IL-10, and PG-E2 that can mediate this immunosuppression [46, 47]. Also it was demonstrated recently that lactic acid which is extensively produced by major of tumors is potent immunosuppressant, metabolites of tryptophan cause potent immunosuppressive microenvironment in gliomas, and nitric oxides also are produced in tumors and cause immunosuppression [48–51]. High systemic levels of these compounds in organism during tumor last stage expansion could cause systemic toxic effects and lead to systemic immunosuppression as it was found in our experiments in symptomatic mice. We assume that this systemic immunosuppression may be a result of toxic stress syndrome, and corticosteroid hormones could play a key role in development of systemic immunosuppression. However, recent reports address inadequacy of murine models of human diseases, especially inflammatory diseases and cancer and extrapolation of these results to a human brain cancer should be done with caution.

## Abbreviations

As: Asymptomatic
BBB: Blood brain barrier
CNS: Central nervous system
HBSS: Hanks buffered salt solution
HA: Hemagglutinin
HA-Vac: HA vaccinia virus
i.c.: Intracranial
Sy: Symptomatic
TCR: T-cell receptor for antigen
Th: T helper.
